# Strengthening population and organizational health literacy to reduce social inequalities within the JA PreventNCD

**DOI:** 10.1177/14034948251372119

**Published:** 2025-09-09

**Authors:** Christopher Le, Christa Straßmayr, Nicolas Giraudeau, Johanna Cresswell-Smith, Camille Barailla, Peder Ringnes Berrefjord, Pia Solin, Raffaella Bucciardini, Anita Thorolvsen Munch, Robert Griebler

**Affiliations:** 1 Norwegian Directorate of Health, Oslo, Norway; 2 University of Inland Norway, Faculty of Social and Health Sciences, Elverum, Norway; 3 Austrian National Public Health Institute, Vienna, Austria; 4 Centre Hospitalier Universitaire de Montpellier, France; 5 Finnish Institute for Health and Welfare, Helsinki, Finland; 6 Italian National Institute of Health, Rome, Italy

**Keywords:** Health literacy, digital health literacy, organizational health literacy, mental health literacy, Theory of Change, JA PreventNCD

## Abstract

**Aims:**

Health literacy is considered a key social determinant of health. It plays an important role in the prevention and management of non-communicable diseases (NCDs), contributing to better health and well-being. Therefore, the overall aim of the health literacy focus (Action) within the European Joint Action to prevent NCDs (JA PreventNCD) is to promote general, digital, mental and organizational health literacy to improve health outcomes and counteract NCD-related health inequities in Europe. This paper presents a rational and methodological approach for strengthening health literacy in Europe.

**Methods:**

The Action will be implemented through collaboration among 13 EU Member States and three non-EU Member States, using both quantitative and qualitative research approaches including questionnaire-based surveys, literature reviews, in-depth interviews, and focus groups, as well as stakeholder and user involvement. The implementation will be guided by a Health Literacy Approach to Theory of Change and carried out in three main phases: 1) project development, 2) data collection, data-analysis and tool development, and 3) recommendations for policy, research, and practice and dissemination.

**Conclusions:**

**The project supports participating countries in conducting a comprehensive national-level situation analysis and provides a solid foundation for further developing national health literacy-related policies, strategies or action plans. The project’s expected outcome will contribute to promoting general, digital, mental and organizational health literacy in the prevention of NCDs in Europe. A Theory of Change and a coordinated approach facilitate the development of shared platforms, such as the European Health Literacy Arena, which help maintain collaboration and visibility beyond the lifetime of the project.**

## Background

Non-communicable diseases (NCDs) account for almost three-quarters of the global disease burden and constitute the leading cause of mortality worldwide [1]. In the World Health Organization (WHO) European Region, the five major NCDs (diabetes, cardiovascular diseases, cancer, chronic respiratory diseases, and mental disorders) account for 86% of deaths and 77% of the disease burden [2]. These diseases are linked to lifestyle, working and living conditions and healthcare quality, making them a significant yet largely preventable health issue in Europe.

Health literacy is considered a social determinant of health [3], which is crucial for health, influencing behaviours, coping with social determinants, and healthcare service use. Studies indicate that low health literacy is linked to unhealthier lifestyles, poorer health outcomes, higher mortality [4–11], increased use of healthcare services and ultimately higher healthcare costs [4,10,12], estimated at 3–5% of national health budgets [13]. Recognizing the importance of health literacy, many countries and international organizations such as the WHO and the European Union (EU) have adopted strategies to enhance health literacy [14,15].

According to the WHO [16], ‘Health literacy represents the personal knowledge and competencies that accumulate through daily activities, social interactions and across generations.’ It is also influenced by ‘. . .organizational structures and availability of resources that enable people to access, understand, appraise and use information and services in ways that promote and maintain good health and well-being for themselves and those around them.’ The COVID-19 pandemic highlighted the importance of health literacy, as disinformation and misinformation spread rapidly [17], impacting the prevention and control of NCDs, and worsening health inequities [18,19]. In light of this, new interventions have been developed for disseminating trustworthy health information, although people with low health literacy have been under-represented in these efforts [20–22]. The United Nations (UN) and the WHO emphasize that limited health literacy might be linked to health disparities and social inequalities [23,24]. Individuals with low socio-economic backgrounds or chronic health conditions tend to have poorer health literacy [9,25] and are less likely to use primary healthcare [9,26] and preventive services, which may lead to delayed diagnosis and treatment, increasing the risk for undertreatment and rehospitalization [4]. Among these, people with a migrant background – which is itself recognized as a social determinant of health – are particularly vulnerable [27–29].

Mental disorders are a leading cause of disability and a significant risk factor for premature mortality [30,31]. A recent study estimated the global economic burden of mental disorders to be approximately USD5 trillion [32], highlighting the need for a greater focus on mental health within NCDs. Mental health literacy (MHL) is gaining attention in this respect, defined by Kutcher et al. [33] as (i) understanding how to obtain and maintain positive mental health; (ii) understanding mental disorders and treatments; (iii) reducing stigma and (iv) enhancing help-seeking efficacy. Higher MHL levels are linked to positive health behaviours [34], more supportive attitudes towards mental health problems, and increased help-seeking behaviours [35]. Improving MHL can enhance understanding and support for both mental illness and positive mental health [36], when following a dual continuum approach in which flourishing (i.e. positive mental health) and mental illness are viewed as distinct but related concepts [37].

The digitization of public health and healthcare services requires a high level of digital health literacy (DHL) among users [38], which is defined as ‘the ability to search for, access, understand, appraise, validate and apply online health information, and the ability to formulate and express questions, opinions, thoughts, or feelings when using digital devices’ [9,39]. A recent study found low DHL among young people aged 16 to 25 years, with difficulty in accessing quality health information [38]. Higher DHL levels have been linked to better navigation of the healthcare system. It encompasses the individual, social and technical skills needed to effectively use digital health information and services [9,40], which can support disease prevention and the promotion of health and well-being [38].

Recent research on health literacy has shifted from focusing solely on individual skills to recognizing the influence of its contextual factors. Health literacy involves health-related decision making which is often beyond an individual’s control [41]. Parker [42] emphasized it as a relational concept in which individuals’ skills must match the demands of their specific health information and service environment. This approach, known as organizational health literacy (OHL), suggests that health services must be responsive to varying levels of health literacy, making it a more effective strategy than targeting individuals’ skills alone [43,44]. OHL in healthcare organizations has been defined as the degree to which healthcare organizations equitably enable people, through organizational structures, policies and processes, to find, understand, appraise and use information and services to make health-related decisions and actions for themselves and others [45,46]. In other words, the concept of OHL promotes the responsibility of healthcare organizations to ensure that people’s health literacy needs and preferences are met [42,47–49]. Specifically, Brach et al. [50] described in their report in 2012 that ‘a health literate organization is one that supports low literate patients to navigate, understand, and use information and services to take care of their health’.

The WHO Action Network on Measuring Population and Organizational Health Literacy (M-POHL) aims to enhance health literacy in the WHO European Region by providing high-quality, internationally comparative data to support evidence-based decisions and targeted interventions (m-pohl.net). Following its first project, the Health Literacy Survey 2019–2021 (HLS_19_), M-POHL continues its focus on strengthening OHL within healthcare organizations (OHL project) [51]. The OHL project aims to create health-literate organizations to help users, patients, employees, managers and citizens access, navigate, understand, appraise and use health information and services [50]. Other ongoing M-POHL projects include the EVPOP project on Evidence-based Policy and Practice, and the Health Literacy Survey 2024–2026 (HLS_24_). M-POHL is, therefore, an important strategic partner for the European Joint Action on Cancer and other NCDs prevention – Action on Health Determinants (JA PreventNCD) [52].

**Figure 1.**
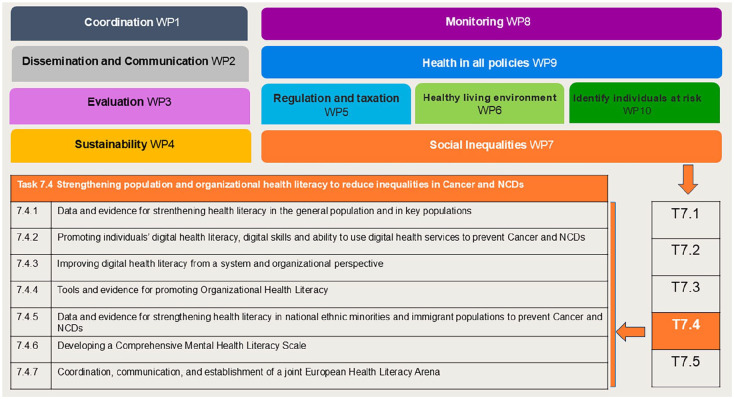
Task 7.4. Strengthening population and organizational health literacy to reduce social inequalities within the JA PreventNCD. JA PreventNCD: Joint Action to prevent NCDs; NCD: non-communicable disease.

This conceptual paper outlines the objectives and methodological approaches of the specific health literacy focus (hereafter called the ‘Action’) within the broader context of addressing social inequalities within the JA PreventNCD project (Figure 1).

## Aim and specific objectives

The overall aim of the Action is to promote general, digital, mental and organizational health literacy in Europe, striving to counteract health inequities in NCDs. This is operationalized through five main objectives: (i) to identify general and digital health literacy gaps and challenges in the general population and key populations, (ii) to provide insights into existing evidence and effective measures to improve general and digital health literacy of populations and the health literacy responsiveness of healthcare services, (iii) to improve the health literacy responsiveness of healthcare services by supporting the implementation of OHL standards, (iv) to develop a comprehensive MHL scale which considers both continua of mental health and (v) to enhance awareness and responsiveness to health literacy at the European, national and subnational levels.

## Methodological approaches

**Figure 2.**
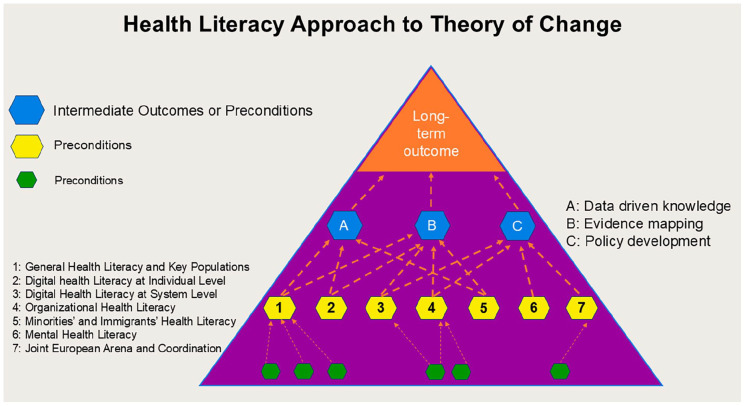
Health Literacy Approach to Theory of Change.

The Action aims to lay the foundation for strengthening health literacy as a means of counteracting health inequities in the context of NCDs. It will provide data and evidence and facilitate actions to strengthen health literacy at the population and organizational level, such as facilitating easy access to high quality health information, supporting critical appraisal of digitally available health information, helping people navigate the healthcare system, learning about the challenges using health information, and promoting the health literacy responsiveness of healthcare organizations. It will also contribute to developing a new MHL scale for comprehensively measuring both continua of mental health. As such, a Health Literacy Approach to Theory of Change will be applied to guide this Action and outlines the specific activities intended to achieve preconditions and long-term outcomes, serving as the foundation for the project’s concrete activities. It is expected that the long-term outcomes of this Action will strengthen different aspects of health literacy in various populations, thereby contributing to the reduction of social inequalities in health. The underlying Theory of Change is defined as ‘a particular approach for making underlying assumptions in a change project explicit and using the desired outcomes of the project as a mechanism to guide project planning, implementation, and evaluation’ [53]. The Health Literacy Approach to Theory of Change is illustrated in Figure 2 and further described by a logical framework in the Supplemental material Table online.

The Action has a mixed-methods design, applying both quantitative and qualitative research approaches through questionnaire-based surveys, scoping/systematic/umbrella literature reviews, in-depth interviews, focus groups, and stakeholder and user involvement. It will be carried out in three phases: 1) project development, 2) data collection, data analysis and tool development and 3) recommendations for policy, research, and practice and dissemination over the four-year period 2024–2027.

Overall, the Action is led by Norway with 13 EU Member States (Austria, Belgium, Czech Republic, Estonia, Finland, France, Germany, Greece, Hungary, Italy, Lithuania, Poland and Spain) and three non-EU Member States (Iceland, Norway and Ukraine) participating, and one observer (Slovenia). The implementation is organized with seven subtasks as described in the following sections.

### General health literacy in general and key populations

Co-led by Austria and Norway, this subtask aims at collecting data and evidence for strengthening health literacy in the general and in key populations by:

- Carrying out a representative population health literacy survey in (most of) the participating countries to identify health literacy challenges, ideally as part of the HLS_24_ M-POHL project;
- Investigating health literacy gaps between population groups to identify specific target groups;
- Mapping existing evidence on effective interventions to improve health literacy in the population or in specific population groups by conducting a scoping review following with a systematic review;
- Collecting models of good and promising practice from participating countries;
- Reviewing promising interventions by involving users and people with lived experience.

### DHL, digital skills and ability to utilize digital health services in the general population

Coordinated by France with support from Austria and Norway, this subtask seeks to:

- Measure DHL in the population to identify digital health literacy challenges and gaps, ideally as part of the HLS_24_ M-POHL project;
- Map existing evidence on effective interventions to improve DHL in the population or in specific population groups;
- Review promising interventions by involving users.

### DHL from a system and organizational perspective

Led by France with support from Norway, this subtask aims to:

- Identify technology, infrastructure, and human capital resources available (and those lacking) to support DHL in the population;
- Identify competencies and capacities necessary to support DHL; competencies should be assessed at societal, organizational and individual levels;
- Adopt the IDEAHL European Digital Health Literacy Strategy to address any gaps identified.

### OHL

Co-led by Austria and Norway, this action aims to:

- Develop an implementation strategy to engage primary healthcare organizations and hospitals in assessing their health literacy responsiveness;
- Pilot self-assessment OHL-tools for primary healthcare and hospitals;
- Conduct OHL assessments in healthcare organizations to identify OHL challenges, ideally as part of the M-POHL OHL-project;
- Map existing evidence and effective interventions to improve the OHL of healthcare organizations;
- Review identified interventions by involving users;
- Develop an OHL toolkit to support healthcare organizations in becoming health-literate organizations.

**Figure 3.**
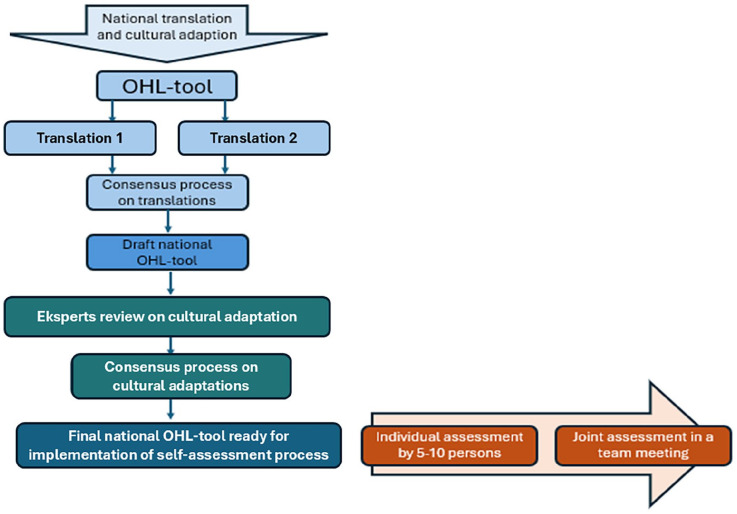
Standardized procedure of M-POHL for translation, cultural adaptation, and assessment process using organizational health literacy (OHL) tools. M-POHL: Action Network on Measuring Population and Organizational Health Literacy.

This subtask is being conducted in cooperation with M-POHL [54], from which participating countries will receive a tool for assessing OHL either in hospital or in the primary healthcare settings. Translation of the OHL self-assessment tool into national languages will follow M-POHL’s standardized procedure for translation and cultural adaptation, as shown in Figure 3.

The self-assessment process involves two parts, in which (i) 5–10 recruited participants perform and fill in the OHL-tool following with (ii) a joint assessment conducted by a team that discusses results from the individual assessment that forms the basis for identifying areas for improvement and next steps on becoming a health-literate healthcare organization.

### Health literacy in national ethnic minorities and immigrant populations

Coordinated by France with support from Austria and Norway, this subtask will be carried out by:

- Measuring health literacy in ethnic minorities and immigrant populations;
- Mapping existing evidence and effective interventions to improve health literacy in ethnic minorities and immigrant populations, reviewing interventions by involving users;
- Piloting selected interventions and evaluating their implementation;
- Seeking synergies with relevant pilots within the WP7 Social Inequalities.

### MHL

Led by Finland, this subtask aims to develop a comprehensive MHL scale by:

- Developing a conceptual model for comprehensive MHL;
- Item selection via deductive (i.e. literature review and assessment of existing scales) and inductive methods (i.e. exploratory research methodologies including focus group discussions and interviews);
- Piloting the comprehensive MHL scale in partner countries in a variety of community settings (adult population);
- Evaluating and amending the scale according to pilot test results;
- Validating the scale in different countries and settings.

### Joint European Health Literacy Arena and coordination

Led by Norway, this subtask aims to establish a joint European Health Literacy Arena for sharing experiences and disseminating and promoting best practices. Effective coordination is essential to ensure that the different strands of work on health literacy within the Action are aligned, complementary and mutually reinforcing. Given the diversity of focus areas – ranging from DHL and OHL to MHL and focus on vulnerable groups – structured collaboration helps identify shared challenges, avoid duplication and promote joint solutions. Activities include:

- Ensuring coherence across the health literacy actions within the JA PreventNCD and aligning with other relevant European Joint Actions, such as Joint Action Cardiovascular Diseases and Diabetes (JACARDI) and Joint Action on Cancer Screening (EUCanScreen), to advance health literacy;
- Facilitating networking, and engaging and activating experts, stakeholders and users across non-governmental organizations, policymakers, healthcare services and academic realms;
- Arranging 2–3 annual virtual meetings/conferences and one in-person annual task meeting.

## Discussion

Health literacy in the JA PreventNCD initiative has been framed within the broader understanding of literacy and is considered a key social determinant of health [3]. Although research directly linking health literacy and social inequalities in health remains scarce, efforts to increase population health literacy are seen as a critical part of the work to reduce social inequalities [55]. Accordingly, expected results from this Action will illustrate gaps between population groups and evidence on effective interventions or best practices. Increasing population health literacy can help strengthen people’s abilities to cope with various life situations [56], including both physical and mental health aspects.

As factors outside the health sector, such as everyday aspects of our daily lives [57], have a strong influence on population mental health, it is imperative that we are able to measure MHL in a broader sense, including both positive mental health (mental wellbeing) and mental health difficulties. The development of a comprehensive MHL scale is expected to benefit broader measurement of how mental health is understood.

Even when health services are available, people are not always able to access the services they need, which has been linked to low health literacy [58]. Identified technology, infrastructure and human capital resources available and the competencies and capacities necessary to support DHL and health literacy at a societal, organizational and individual level is an expected outcome from this joint Action. Furthermore, OHL assessment tools and an OHL toolkit should encourage healthcare organizations to become more health literate and accessible to people with low health literacy. Enhancing OHL can improve healthcare organizations’ responsiveness to the needs of patients with NCDs, thereby contributing to the reduction of health inequalities.

A high level of health literacy and DHL is linked to sound physical and mental health, while a low level can constitute a societal and public health burden [26,38,59,60]. Health literacy plays an important role in combating NCDs and antibiotic use and resistance, as well as addressing the social determinants of health [2,7,9,15,55,61]. MHL, on the other hand, is needed for addressing mental health as an NCD in its own right, as well as a comorbid factor to other illnesses [59,60]. Subsequently, improving health literacy and MHL will be essential to the sustainable use of health resources across the life-course and enable health authorities to better prevent harm and disease, increase the effectiveness of health promotion initiatives and reduce healthcare expenditures. Increasing population health literacy could therefore reduce the burden on the healthcare system and help improve the strained post-COVID-19 healthcare situation. Given the high burden of mental illness, attention to MHL is also timely.

Conducting national health literacy surveys provides an overview of the population’s health literacy, including areas that need further attention, and contributes to enhancing insights for strengthening health literacy. The surveys provide data-driven support for further monitoring and evaluation of health literacy initiatives related to high-risk groups in action plans and strategies. An overview of promising health literacy interventions enables countries to make decisions about the systematic implementation of effective health literacy interventions.

Accessing, understanding, navigating and using the healthcare system is difficult for almost everyone. This is even more challenging for people with an immigrant or ethnic background, a different culture (different understanding of the health system, healthcare and illness) and limited language proficiency [62]. To leave no one behind, all research and implementation activities must include relevant groups that are particularly vulnerable. Evidence-based information on health literacy in different populations is therefore essential for tailoring health information, services and interventions. It is also important for empowering individuals to take an active role in their own health.

While low health literacy is associated with several adverse health outcomes, such as the incidence of chronic disease [9], healthcare organizations play a critical role in promoting health literacy in the community or among patients by helping them navigate the healthcare system and to self-manage chronic conditions [63,64]. Health-literate healthcare organizations make it easier for all stakeholders (patients/relatives, staff/management and citizens) to access, understand, appraise and use disease- and health-related information and services. They aim to improve the professional health literacy of healthcare professionals to strengthen the health literacy of their patients so that they can make better everyday decisions about their healthcare (co-production), disease prevention and health promotion [65,66]. This Action will support the implementation of internationally developed standards for assessing OHL in both primary healthcare and hospitals and will seek to provide support on how healthcare organizations can become more health-literate [67]. By focusing on improving OHL, healthcare organizations will become more responsive to the needs of patients with NCDs. Consequently, WP2 (Dissemination and Communication) within the JA PreventNCD takes into account health literacy and OHL as and among success factors for the project’s visibility, impact and stakeholder engagement.

Health literacy is closely connected to many of the priorities addressed across the Joint Action. Strengthening people’s ability to find, understand, appraise and apply health information can support prevention efforts, improve access to services and enhance user involvement – all of which are critical for reducing inequalities in NCDs. Several pilot activities, for example in WP10 (Identify Individuals at Risk), touch on these themes, including efforts to improve communication in primary care, adapt self-management tools for low-literate populations and test digital health solutions for underserved groups. In WP7 (Social Inequalities), a dedicated cluster of pilots focuses specifically on health literacy-related challenges, offering practical examples of how the concept can be applied to reduce complexity in health systems and improve outcomes for vulnerable groups. Ongoing collaboration and exchange across work packages help ensure that insights from the health literacy work are relevant and taken up in other parts of the Joint Action. In addition, links with other European initiatives, such as the JACARDI Joint Action, further reinforce the potential for joint learning and shared impact.

The international HLS_19_ study showed a differing degree of social gradient for all participating countries, whereas financial deprivation and self-perceived level in society were the strongest predictors for all aspects of health literacy [9]. Consequently, improving population health literacy is an intervention that can contribute to reducing social inequalities in health by empowering individuals to have greater control over social determinants of health. However, health literacy interventions are not a universal solution to social inequalities in health and do not replace the need to implement other interventions that address the root causes of social health inequalities [3,68]. Marmot and Bell refer to these root causes as ‘the causes of the causes’ [69] and believe that interventions should address the social determinants of health that result from the unequal distribution of power, resources and opportunities [3,68].

Countries participating in this Action have the opportunity to contribute to the strengthening of the international database on health literacy in Europe. Moreover, this project will support participating countries to continue to gain a holistic status quo analysis, which is recommended as the first step in the M-POHL guide for policy and decision makers in developing and implementing national health literacy strategies [70]. This provides a solid basis for further development of national health literacy policies, strategies or action plans. In order to achieve a healthier population, it is essential that health authorities learn and take into consideration how the public find, understand, appraise and apply health information. Accordingly, WP10 (Identify Individuals at Risk) within the JA PreventNCD considers health literacy as a risk factor of effective prevention of NCDs. To take concrete action in strengthening and promoting health literacy and thereby reducing inequalities in NCDs, five pilot actions have been planned across five European countries. These initiatives are part of WP7 (Social Inequalities) within the JA PreventNCD project. A group of experts specializing in social inequalities will support each action. They will employ appropriate methodologies to monitor progress and evaluate the final outcomes using both outcome and impact indicators.

Finally, a joint European Health Literacy Arena will foster further collaboration, facilitate knowledge translation and increase the impact of health literacy initiatives. By harmonizing and communicating all activities, this component will support the implementation and dissemination of the health literacy strategies developed within JA PreventNCD across the different phases of the project.

To evaluate complex community initiatives aimed at driving social change, a Health Literacy Approach to Theory of Change was developed as a tool to clearly outline underlying assumptions from the outset [71]. The process of creating a Theory of Change enables a project team to build consensus around these assumptions, which are then formalized into a tangible output. This output is tailored to the specific context of the initiative. Instead of merely asking ‘does it work?’, the goal of this approach is to explore ‘under what conditions does something work, and for whom?’ [72]. Therefore, developing a Health Literacy Approach to Theory of Change gives stakeholders the opportunity to critically examine the logic, linking preconditions to planned activities while the project is still in its planning stages. Furthermore, the process of crafting and evaluating a Theory of Change compels stakeholders to clearly define how resources will be utilized to achieve the preconditions necessary for the long-term goal they aim to accomplish.

## Conclusion

Overall, the health literacy focus within the JA PreventNCD represents a unique opportunity for countries within Europe to collectively put health literacy, DHL, MHL and OHL on the policy, practice and research agenda for the prevention of NCDs. Enhancing individuals’ ability to find, understand, appraise and apply health information can bolster prevention efforts, improve access to healthcare services and promote active user involvement. These are key factors in reducing health inequalities and preventing NCDs.

Generating data to identify health literacy gaps and challenges in the populations, developing measurement and assessment tools, and disseminating promising and evidence-based practices will support countries’ health literacy policy development. In addition to supporting synergies across tasks and partners, coordination also helps maintain attention on important cross-cutting issues such as DHL and intersectoral collaboration.

Coordination activities such as regular exchanges, shared planning tools and joint dissemination efforts enable partners to learn from each other and build on each other’s work. This not only strengthens the internal coherence of the Action but also increases its relevance and potential impact across countries and policy areas. A Theory of Change and coordinated approach also supports the development of shared platforms, such as the European Health Literacy Arena, to help maintain collaboration and visibility beyond the lifetime of the project.

## Supplemental Material

sj-docx-1-sjp-10.1177_14034948251372119 – Supplemental material for Strengthening population and organizational health literacy to reduce social inequalities within the JA PreventNCD Supplemental material, sj-docx-1-sjp-10.1177_14034948251372119 for Strengthening population and organizational health literacy to reduce social inequalities within the JA PreventNCD by Christopher Le, Christa Straßmayr, Nicolas Giraudeau, Johanna Cresswell-Smith, Camille Barailla, Peder Ringnes Berrefjord, Pia Solin, Raffaella Bucciardini, Anita Thorolvsen Munch and Robert Griebler in Scandinavian Journal of Public Health
