# Supplementary material for: Strengthening population and organizational health literacy to reduce social inequalities within the JA PreventNCD
Source: Scand J Public Health. 2025 Sep 9;53(3 Suppl):43–53. doi: 10.1177/14034948251372119 (PMC12708958; doi:10.1177/14034948251372119)
Supplement: sj-docx-1-sjp-10.1177_14034948251372119 – Supplemental material for Strengthening population and organizational health literacy to reduce social inequalities within the JA PreventNCD [file sj-docx-1-sjp-10.1177_14034948251372119.docx]

# Health Literacy Approach to Theory of Change – Logical Framework

| **Level** | **Result** | **Indicator** |
| --- | --- | --- |
| Overall objective (Impact) | To contribute to the reduction of avoidable inequalities in cancer and NCDs across European countries | Within 5 to 10 years, observable reduction in measurable disparities in cancer and NCD outcomes (e.g. incidence, mortality, screening uptake) across and within participating countries, stratified by socioeconomic, geographic, or demographic variables." |
| Specific objective (outcome) | To contribute to the generation of actionable evidence on population health literacy and the responsiveness of health care services to support improvements in policy and practice in JA PreventNCD participating countries. | At least 70% of the countries participating in Task 7.4 generate actionable evidence on general, digital, organizational, and mental health literacy , including among vulnerable groups and transfer such evidence into policy tools, recommendations, action plans or official documents.” |
| Intermediate objective (outcome) | Increase knowledge of: a) Health literacy (HL) challenges in populations and key groups. b) Existing promising interventions that address HL challenges. | - At least 60% of the countries participating in this sub-task collect HL data and publish a national report. - At least 60% of the countries participating in this sub-task share HL findings and recommendations with relevant policy makers to inform national strategies or planning documents. |
| Intermediate objective (outcome) | Improve individuals' digital health literacy (DHL) by identifying gaps, effective interventions, and models of good practice. | - At least 50% of countries participating in this sub-task collect and disseminate examples of effective DHL interventions. - At least 60% of countries participating in this sub-task produce policy recommendations to improve individual DHL. |
| Intermediate objective (outcome) | Strengthen system and organizational digital health literacy (DHL) by identifying key resources and competencies and co-creating a DHL-specific action plan. | - At least 30% of countries participating in this sub-task carry out a system-level mapping of DHL competencies and resources. – - At least 40% of countries participating in this sub-task co-develop a DHL-specific action plan through a participatory process. |
| Intermediate objective (outcome) | Enhance organizational health literacy (OHL) in health care services by supporting the implementation of standards and improving responsiveness to patient needs. | - At least 80% of countries participating in the sub-task conduct OHL assessments using validated tools. – - At least 80% of countries participating in this sub-task involve relevant stakeholders from healthcare organizations to raise awareness of OHL. |
| Intermediate objective (outcome) | Address health literacy challenges in ethnic minority and immigrant populations by measuring HL levels, identifying HL gaps, and mapping effective interventions. | - At least 60% of countries participating in this sub-task conduct HL assessments targeting migrants or ethnic minority groups. - At least 40% of countries participating in this sub-task produce policy briefs or guidelines promoting inclusive HL strategies. |
| Intermediate objective (outcome) | Develop and promote approaches to measuring mental health literacy that also incorporate positive aspects of mental health. | - At least 70% of countries participating in this sub-task contribute to the development of the new mental health literacy scale. - At least 70% of countries participating in this sub-task attend a consultation or workshop on piloting or adopting HL tools, including those for mental health |
| Intermediate objective (outcome) | Enhance awareness of and responsiveness to health literacy at the European, national, and subnational levels to support policy development and implementation. | - 100% of countries in Task 7.4 participate in at least one annual coordination or knowledge-sharing event under WP7. - At least 70% of countries in task 7.4 reference Task 7.4 findings in official reports, policy papers, or funding proposals. |
